# Magnetic Field (MF) Applications in Plants: An Overview

**DOI:** 10.3390/plants9091139

**Published:** 2020-09-03

**Authors:** Mohammad Sarraf, Sunita Kataria, Houda Taimourya, Lucielen Oliveira Santos, Renata Diane Menegatti, Meeta Jain, Muhammad Ihtisham, Shiliang Liu

**Affiliations:** 1 College of Landscape Architecture, Sichuan Agricultural University, Chengdu 611130, China; sarraf.science@gmail.com; 2 Department of Horticulture Science, Shiraz Branch, Islamic Azad University, Shiraz 71987-74731, Iran; 3 School of Biochemistry, Devi Ahilya Vishwavidyalaya, Indore 452001, India; sunita_kataria@yahoo.com (S.K.); mjjainmeeta@gmail.com (M.J.); 4 Department of Horticulture, Horticol complex of Agadir (CHA), Agronomy and Veterinary Institute Hassan II, Agadir 80000, Morocco; houdataimourya@yahoo.com; 5 Laboratory of Biotechnology, School of Chemistry and Food, Federal University of Rio Grande, Rio Grande-RS 96203-900, Brazil; lucielensantos@furg.br; 6 Department of Botany, Institute of Biology, Federal University of Pelotas, Rio Grande-RS 96203-900, Brazil; renata.menegatti@ufpel.edu.br; 7 College of Horticulture and Forestry, Huazhong Agricultural University, Wuhan 430070, China

**Keywords:** abiotic stress, crop yield, magnetic field, magnetized water, magneto-priming, seed germination

## Abstract

Crop yield can be raised by establishment of adequate plant stand using seeds with high germination ratio and vigor. Various pre-sowing treatments are adopted to achieve this objective. One of these approaches is the exposure of seeds to a low-to-medium level magnetic field (MF), in pulsed and continuous modes, as they have shown positive results in a number of crop seeds. On the basis of the sensitivity of plants to MF, different types of MF have been used for magnetopriming studies, such as weak static homogeneous magnetic fields (0–100 μT, including GMF), strong homogeneous magnetic fields (milliTesla to Tesla), and extremely low frequency (ELF) magnetic fields of low-to-moderate (several hundred μT) magnetic flux densities. The agronomic application of MFs in plants has shown potential in altering conventional plant production systems; increasing mean germination rates, and root and shoot growth; having high productivity; increasing photosynthetic pigment content; and intensifying cell division, as well as water and nutrient uptake. Furthermore, different studies suggest that MFs prevent the large injuries produced/inflicted by diseases and pests on agricultural crops and other economically important plants and assist in reducing the oxidative damage in plants caused by stress situations. An improved understanding of the interactions between the MF and the plant responses could revolutionize crop production through increased resistance to disease and stress conditions, as well as the superiority of nutrient and water utilization, resulting in the improvement of crop yield. In this review, we summarize the potential applications of MF and the key processes involved in agronomic applications. Furthermore, in order to ensure both the safe usage and acceptance of this new opportunity, the adverse effects are also discussed.

## 1. Introduction

Seed germination is enhanced by pre-sowing treatments through chemical or physical methods by breaking dormancy, which protects the seeds against pest and diseases and provides uniform crop stand establishment in the field. Seed vigor and vitality are lost in storage due to deterioration, which ultimately results in loss of expensive seed material. Priming treatments such as osmo-priming, hydro-priming, halo-priming, and solid matrix priming are pre-sowing techniques that hydrate the seeds during the treatment process, thereby improving germination at the cost of seed storage [1,2]. Such seeds cannot be stored for longer periods and have to be sown immediately after the treatment. Keeping this drawback in mind, priming of the seed with a magnetic field (MF) may give a viable alternative for improving seed vigor [1]. During the past decades, the human environment has been changed due to the extreme usage of the MF. The excessive and constant use of household appliances, medical instruments, transportation vehicles, and communication equipment expose plants (more than any other species) to a greater amount of the MF [3]. This is because the intensity of the geomagnetic field that effects plant growth by natural MF with an intensity of 50–60 μT [4] is increasing and the progress in technology changes this MF intensity. Therefore, when exposed to natural MF, plants respond differently on the basis of MF intensity, which may positively or negatively influence their development [5]. The effects of the MF in seeds and plants have made impressive advances in the last 10 years in plant science. Currently, there is real evidence that magnetic pre-germination treatment of seeds before sowing allows for reduced costs of planting as germination rates are increased substantially, as well as the plant growth being promoted [6,7,8]. However, others showed that development is inhibited [9,10].

Various agronomic practices have often been employed to improve the germination pre-treatment of seeds, which stimulates seed germination, emergence, and vigor. This paper highlights the MF as an alternative to conventional treatments based on chemical substances (plant growth regulators), reinforcing the importance of understanding the different interactions between electromagnetic fields and plant physiological processes [11,12,13]. Crop yields in general and the homogeneity are raised with the application of the chemical substances (such as hormones) in the pre-sowing seed treatment, although they are considered very effective and invasive, ecologically incorrect, and difficult to apply. During the last decades, the discovery of hormones and chemical fertilizers played an important yield-enhancing factor in plant cultivation, but the current use of chemical fertilizers and other materials have been controversial, which has directed the use of alternative ecologically friendly treatments with reduced cost, such as gamma rays, laser, electron beam, microwave, MF, and radiofrequency energies to bring about bio-stimulation of seeds [14,15]. Thus, biophysical treatment may be considered as an alternative because it reduces the amount of toxin in plants or products of plants and results in increasing food and environmental safety [14]. Magnetic field treatment of seeds became very popular in the agricultural sector. Pre-sowing seed treatment with MF, called “magnetopriming”, is a non-destructive and dry seed priming treatment that has been reported to increase the rate of germination and seedling vigor of many crops [16,17,18,19]. There are several reports on the metabolic changes occurring during germination in seeds in response to magnetopriming under non-stressed environments [16,17,18,19]. The effects of magnetic bio-stimulation of seeds under salt stress using stationary MF was reported by Thomas et al. [20] and Kataria et al. [21].

Another advantage in the use of MF is in relation to increasing the germination rates and the possible increase in membrane permeability, facilitating the process of water absorption by seeds [22]. There are still some other studies that have confirmed the effect of magnetized water in plants or seeds. In this sense, Maheshwari and Grewal [23] and Hilal et al. [24] stated that MF may affect water and nutrient absorption, as well as improving plant growth. Beyond improving the germination rates, MF-exposed seeds induce positive effects such as increased cell proliferation capacity, which possibly induces plants rapid growth [25]. Other researchers have also reported the positive effect of MF on increasing seed germination, seedling vigor, photosynthetic pigments, the efficiency of photosystem II (PSII), and performance index based on the absorption of light energy, as well as in promoting efficient photosynthesis and mitigating the adverse effects of salt, water, and UV-B stress in soybean (*Glycine max* L. Merr. Var: JS-335) [21,26,27,28].

In some studies, MF could reduce plant oxidative damage, owing to the activities of antioxidant enzymes such as peroxidase, polyphenol oxidase (PPO), superoxide dismutase (SOD), and catalase (CAT) in plant cells. Specifically, MF affected the antioxidant activity and increased the activity of the free radical ions in plants [29,30,31]. Other studies have reported multiple negative effects of MF on plants. These effects include inhibition of the cell’s growth, increase of the free radicals, increase of lignin and suberin on the walls of the cells, and reduction of the seed germination and the growth of the organs [32]. Furthermore, MF proves to be a potential tool in agriculture when used as a pesticide. For instance, Mahajan and Pandey [6] concluded that the alternative use of the MF to the use of insecticides and pesticides is possible, wherein they employed the magneto-priming technique in seeds to prevent agricultural pests and diseases, obtaining a high yield of mung beans after planting. The results were comparable with the application of chemical fertilizers, insecticides, and pesticides in order to protect the plants from the yellow mung bean mosaic virus. The researchers then assumed that magneto-priming is promising, standing out as an efficient, clean, and affordable technique that induces both plant resistance and high productivity. The present review is focused on the application of MF and MW (magnetized water) in seed germination, plant growth, and development of plants and microalgae.

## 2. The Effects of MF Application on Plant Development

### 2.1. Effects of Magnetic Treatments on Seed Germination

Plants that grow on the earth are affected by natural MF with an intensity of 50–60 µT [33]. The pioneering effort for improving the seed yield through exposing it to MF and electromagnetic fields (EMF) has been performed since 1930 [34]. The effect of exposure to a MF on seed germination has been the objective of many studies, and the enhancement of seed germination due to MF exposure has been confirmed by many scientists [34,35]. Mahajan and Pandey [6] evaluated the impacts of the static magnetic fields (SMF) on the germination of mung bean (*Vigna radiata* (Linn.) Wilczek.) and reported a linear increase in the average germination rate, coefficient of germination rate, and water absorption with increasing the MF intensity. Moreover, Menegatti et al. [8] indicated that the exposure of passion fruit seeds to the MF in an isolated way stimulated seed germination, emergence, and vigor. Magnetically treated chickpea (*Cicer arietinum* L.) seeds showed an improvement in seed performance in terms of germination speed and length, and dry weight seedling, and the response varied with field strength and duration of exposure [36]. The same positive effects on seed germination rate and vigor index were found in cucumber (*Cucumis sativus* L. Var. Barsati) seeds, lettuce (*Lactuca sativa* L.) seeds, corn (*Zea mays* L. Var. HQPM-1) seeds, tomato seeds (*Solanum lycopersicum* L. Var. MST/32), and radish (*Raphanus sativus* L.) seeds by certain scholars [17,22,37,38,39]. Moreover, studies have also confirmed that the magnetically treated seeds grow higher and heavier than the control, and even increase water uptake [22]. They also have deeper and more vigorous roots than the control [14,16,19]. Further, Florez et al. [40] observed an increasing rate of elongation of wheat seedling treated by MF. On the other hand, Belyavskaya [12] reported an increase in the intensity and emissions of carbon dioxide (CO_2_) from 70 to 100% when barley (*Hordeum vulgare* L.) seedlings were treated by 10 µT MF. Moreover, Kavi [41] reported that appropriate MF application reduces the potential hydrogen in the cell wall, prevents seed dormancy, influences the meristematic cell metabolism, increases nutrient uptake, and enhances photosynthetic capacity [34,41]. With all these, the exact mechanisms in which MF affect the seed germination is still unknown, with only a few publications on this aspect.

Scientists have found that the MF enhances seed germination by changing the biochemical processes by stimulating activity of proteins and enzymes [30,42]. In fact, MF can interact with internal electric field of biological systems through its resonating behavior. Living cells possess electric charges exerted by ions or free radicals, which act as endogenous magnets and have been involved in the biochemical processes [43]. Thus, external MF treatment increases ion uptake and therefore improves nutrition value [43]. Others studies stated that the MF interacts with ion current in the membrane of the embryo cell. This interaction changes the ion concentration and osmotic pressure provided on both sides of the membrane, thereby changing the relationship between water and seeds [22].

Moreover, recent research by Anand et al. [19] showed that plants responded to varying MFs by altering their gene expression and phenotype. In fact, they reported that RACK1 (i.e., receptor of activated protein kinase C1) and metallothionein play an important role in signal transduction pathway mediated by the reactive oxygen species (ROS) to enhance the speed of germination in magnetoprimed tomato seeds (*Lycopersicon esculentum* L. Mill. var. Pusa Rohini). Racuciu et al. [44] reported that in a study conducted on hairy roots induced by *Agrobacterium* spp. (not real roots and nor root meristem), the exposure of 50 µT MF had a stimulatory effect on increasing fresh weight, the content of photosynthetic pigments, and nucleic acid content, and raised the length of the corn (*Zea mays* L.) seedlings. However, MF treatment with a higher induction (100 to 250 µT) had an inhibitory effect on the aforementioned parameters. The MF intensity and its effect on several species of plants are summarized in Table 1.

Other studies have shown that the SMF causes induced apoptosis in the cells of suspension-cultured tobacco (*Nicotiana tabacum* L. cv. Burley 21), and the reduction of the growth of basil (*Ocimum basilicum* L.) [9,51]. In addition, Kordas [5] reported that MF caused a slight decrease in stem length of wheat, while grain yield and the amount of straw were slightly increased. In contrast to the aforementioned studies, there are some who disagree with MF [12,52]. For example, Ijaz et al. [10] found that wheat seeds cv. NR-234 with low viability (45%), when submitted to magnetization treatments, showed no increase in germination, and that this treatment was insignificant. Moreover, it has been observed that weak MF ranging from 100 to 0.5 µT negatively affects seed germination, seedling growth of the plants, roots, and cell division in the root meristem [53].

### 2.2. MW Effects on Seeds Germination and Plant Growth

It is widely known that adequate water supplies, as well as capacity of plant water uptake and seed quality, are considered as the most important factors for a plant’s growth. This is particularly important with regard to the global demand for more food from lesser water resources [54]. Therefore, a scientific approach is needed to sustain the productivity of agricultural crops. The treatment of water by MF is another special aspect of using MFs and is one of the hopeful physical techniques to enhance water quality and crop productivity. Magnetically treated water can enhance the agricultural production, as well as seed germination [55], accelerating the vegetative growth of seedlings, which also improves the mineral content of seeds and fruits [54]. Magnetic energy could enhance the physical and/or chemical properties of soil and water quality. The exposure of water to a magnetic field results in alterations of its basic properties such as ionic strength, pH, and surface tension force, providing greater movement capacity by intensifying the internal vibration of water molecules, which improve the polarizing effect, resulting in an increase of water uptake into the cell [56]. Kareem [57] evaluated the effect of magnetized water irrigation on soil pH. Their results showed that pH decreased to near neutral levels. This method can decrease crop growing period and saves more irrigation water.

Hirota et al. [58] showed that when cultivated cucumber seeds were irrigated by magnetized water then it results increase of growth in these plants compared to the control plants. In another study, Fernandez et al. [59] reported that the seedlings bred by MW were stronger and healthier by increasing plant water nutrients absorption. The water productivity was increased by 1.65, 1.70, and 1.88 for eggplant (*Solanum melomgena* L. cv. Florida High Bush), tomato (*Solanum lycopersicon* L. cv. Logaen), and faba beans (*Vicia faba* L.cv. Isban), respectively, by the magnetically treated water (MTW). This led to water savings of 11%, 14.2%, and 13.5% for the three crops, respectively, ultimately increasing the net profit. Using magnetic treatment technique, the ratios of the net return per water unit to that of using untreated water were 1.97, 2.45, and 3.0 for eggplant, tomato, and faba beans, respectively [57].

Other studies confirmed the effects of magnetized water, and stated that the amount of phosphorus in citrus leaves increased when treated by MW [24,60,61]. This may affect the absorption of calcium (Ca) and phosphorus (P) in citrus, and because the plants can access them easier, the plant’s growth will be improved [23]. As such, the researchers concluded that MTW alters water relations in grain, and this effect may partly explain the acceleration of seed metabolism and germination ratio [12,14]. According to a report by Ijaz et al. [10], wheat seeds (*Triticum aestivum* L. cv. NR-234) with low viability (45%) when subjected to MW treatments were invigorated. The pH stability of water absorbed by seeds or plants may have been achieved due to the alteration of its ionicity, resulting from the breakage of hydrogen bonds present in the molecule, which allowed water to be present at a higher concentration of free ions. Comparing the components of soils irrigated by magnetized water and soils irrigated by tap water, Noran et al. [62] observed a difference in the concentration of Ca, P, nitrogen (N), potassium (K), sodium (Na), and magnesium (Mg). They stated that MW decreases the downward mobility of the mineral compounds, which is due to the accelerative crystallization process and deposition of the mineral elements [54]. Maheshwari and Grewal [23] reported the effects of MW in the reduction of the pH in the soil, which resulted in higher nutrient uptake. Where there was an increase of concentrations of Ca and P in peas (*Pisum sativum* L.) and celery (*Apium graveolens* L.), there was a loading restriction of Na, as well as a reduction of toxicity and Na concentration in the aerial parts. Additionally, the magnetic technique allows us to use salty water (salt content of 2000 ppm and up to 5000 ppm) efficiently for irrigating crops [54]. Therefore, Hasan et al. [63] reported that using MW in two *Moringa* species (*Moringa oleifera* Lam. and *Moringa peregrina* (Forssk.) Fiori), cultivated under water saline stress, allowed the recovery of growth inhibition induced by water restriction, chlorosis, and ion disruption. Moreover, some researchers revealed a notable response to stress when irrigating *Silybum marianum* (L.) Gaertn. plants by magnetically treated sea water [64]. MF treatment will decrease harmful effects of salinity stress at early seedling stage and reduce the oxidative damage, leading to improvements in physiological attributes for plant growth under seawater irrigation stress [64]. Bagherifard and Ghasemnezhad [65] stated that the exposure of saline water enhanced different growth criteria of artichoke (*Cynara scolymus* L.) leaves. Other researchers observed that an increase in the germination rate of lettuce seeds (*Lactuca sativa* L.) treated with static MF (0–10 mT) was consistent with the rate of the absorbed water of the seeds [66]. The method of magnetically treated water in soil and plants and its effects on several plants are summarized in Table 2.

### 2.3. Effects of MFs on Reducing Oxidative Damage

Numerous researchers have revealed the effects of MF on the activities of the antioxidant enzymes such as peroxidase (POD), polyphenol oxidase (PPO), superoxide dismutase (SOD), and catalase (CAT) in plant cells. In this respect, Bhardwaj et al. [17] observed an increase in the activities of antioxidant enzymes, namely, SOD by 8%, CAT by 83%, and glutathione reductase (GR) by 77% in cucumber (*Cucumis sativus* L.) seeds exposed to a SMF, when compared to the control. A similar experiment performed on several plant species, including artichoke (*Cynara scolymus* L.) and *Zea mays* L. (genotype = single cross 704), reported that exposure to MF caused a significant increase in the activities of CAT and ascorbate peroxidase (APX) [65,70]. Hajnorouzi et al. [71] concluded that the pretreatment by alternative MF promoted the growth of maize seedling by alleviation the excess production of reactive oxygen species. The activity of SOD in the magnetically treated seedlings decreased, while the total antioxidant capacity of these seedlings increased when compared to the control.

It was revealed that, like human and animal cells, plant cells can be influenced by MF. Moreover, the effects of MFs have been related to uncoupling of the free radical process in membranes [29,30,31]. Moreover, lipids in the cell membranes are prone to oxidative damage because some free radicals tend to concentrate on the membrane and cause oxidative damage known as “lipid peroxidation” [72,73,74,75]. Accumulation of these free radicals can cause oxidative stress [32,53,76], and the oxidative stress causes a change in the enzymatic activity, gene expression, and the release of calcium from intracellular stores. Moreover, this stress can affect the membrane structure and cell growth, and induce cell death (apoptosis) [71,77,78]. By treating soybean seeds by SMFs, Shine and Guruprasad [79] observed an enhancement in the production of the ROS mediated by cell wall peroxidase, while the production of ascorbic acid content, and SOD and APX activities were decreased in the hypocotyl part of the germinating seeds. Numerous experiments performed on several plant species, including soybean (*Glycine max* L. Merr. Var: JS-335), corn (*Z. mays* L. Var. HQPM-1), and tomato (*L. esculentum*) reported that pretreatment with MF induces greater plant resistance later under water stress conditions, suggesting that this efficiency is related to the improvement of the antioxidant system [26,37,80]. Shabrangi and Majd [81], investigating the effects of drought stress in lentil (*Lens culinaris* Medik.) seeds, observed that there was more resistance in seedlings pretreated by magnetic fields, with a significant increase in the APX and SOD activities in both roots and shoots. These antioxidant enzymes scavenge the ROS and other chemical changes produced in the cells under stress. The results suggest that seeds pretreated by MFs allow them to overcome harmful environmental factors. Çelik et al. [82] investigated two enzymes of the defense system, the SOD and CAT activities under MF application. The results indicated that the function of defense enzymes in seedlings was intensified due to the treatment by the MF, which indicated that for plant cells, an MF creates a stress condition similar to other environmental stress factors. However, the increases in the MF exposure times do not cause linear increases in enzyme activities in in vitro and in vivo studies.

Similarly, some studies have reported negative effects of MF on plants. Roux et al. [83] exposed tomato plants to MFs for a short period (10 min), and within minutes of electromagnetic stimulation, stress-related mRNA (calmodulin, calcium-dependent protein kinase, and proteinase inhibitor) accumulated rapidly, and 30 min after the electromagnetic treatment, ATP concentration and adenylate energy charge were transiently decreased. This strongly suggests that they are a direct result of the application of MF, and that this radiation is considered a harmful stimulus by plants. These contradictory outcomes from these studies can be dependent on the characteristics of the field’s exposure, such as intensity and duration.

### 2.4. Alleviation of Abiotic Stresses

Although abiotic stresses, such as salinity, UV-B, and water stress, reduce seedling vigor, germination rate, nodulation, biomass growth rate, carbon, and nitrogen metabolism, all of which decrease crop yield, it has been proven that SMF-treated plants exhibit compensatory performance on all these parameters against abiotic stresses as well as in non-stress conditions [1]. El-Yazied et al. [80] obtained a significant increase in the germination percentage and a reduction in the time needed for germination in tomato. They also observed, in seedlings derived from treated seeds, an increase in stem length, stem diameter, leaf area, and fresh and dry weight, even under saline conditions. Several studies have shown that SMF can increase the seed germination and seedling vigor under salt and heavy metal stress in chickpea, soybean, barley, mung bean, and maize [20,21,47,84,85].

Similar results obtained by Baghel et al. [26] demonstrated the effectiveness of magneto-priming, positive results on plant growth attributes, number of root nodules, nodules, fresh weight, and biomass accumulation in soybean (*Glycine max* L. Merr. Var: JS-335), either exposed or unexposed to saline stress. The results further suggest that the use of MF increased carbon and nitrogen metabolism and improved soybean yield in terms of pod number, seed number, and seed weight under saline and non-saline conditions. Baghel et al. [86], evaluating the effect of SMF on morphological and physiological responses of soybean to water stress, concluded that pretreatment of seeds by MF results in increased photosynthetic pigments, efficiency of PSII, and performance index based on absorption of light energy, and promoted efficiency of photosynthesis and mitigated the adverse effects of water stress in soybean. Further SMF pre-treatment enhanced the efficiency of PSII, rate of photosynthesis, and crop yield under abiotic stresses such as UV-B and salt stress by decreasing ROS [27,28,87]. Anand et al. [87] explained the alleviation of adverse effects of water stress by the fact that MF reduced free radical productions and antioxidant enzyme activity. Adaptive response of plants by magnetopriming under abiotic stress in several plant species are summarized in Table 3.

**Figure 1:**
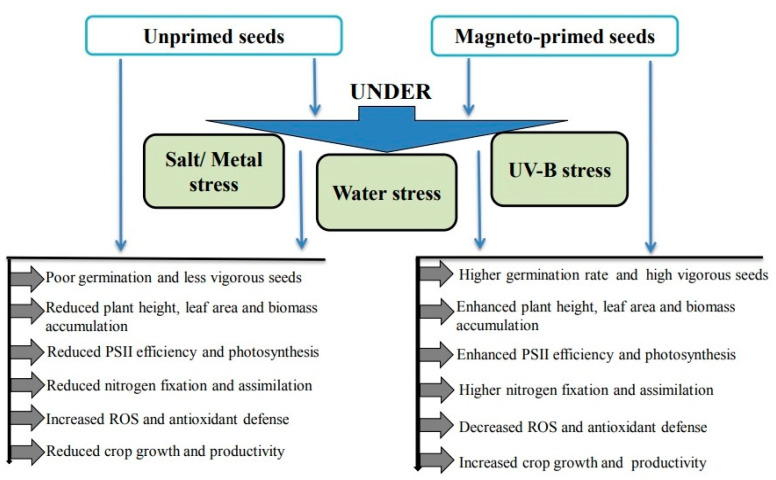
The effect of magnetic field treatment on plants’ abiotic stresses.

Pre-treatment with SMF in soybean exposed to salt stress also had a positive effect on increasing α-amylase, protease, and NR activities, along with higher levels of H_2_O_2_, O_2_^•−^, and nitric oxide (NO) [45]. The authors have suggested NO as one of the main signaling molecules in MF-induced salt tolerance in soybean seedlings. We tried to clarify how magnetic field alleviates abiotic stresses in plants (Figure 1).

## 3. The Effects of MFs on Microalgae

Microalgae are microorganisms that may be prokaryotic or eukaryotic, just as plants are photosynthetic living beings. Several studies have verified the influence of the MF application during crops and positive or negative results in growth or production of compounds has been observed [89]. In microalgae, these effects depend on the physiological state of the cell, cell type (prokaryotic or eukaryotic), exposure time, intensity, form of application, and type of field-generating device. Some authors report possible explanations of how the MF affects the microalgae growth, and since 2010, the number of studies has increased. Small et al. [90] cited that MF alters the concentrations of free radicals due to increased oxidative stress, with this being the most likely mechanism for the effect of MF on microalgae. Beruto et al. [91] concluded that the low frequency electromagnetic fields (EM-ELF) did not act on the mitotic division, but played a significant role in promoting cell clusterization in the liquid phase. Wang et al. [92] observed that MF increases the microalgae growth and regulates its antioxidant defense system to protect cells efficiently. Tu et al. [93] verified that MF stimulates algal growth and oxygen production using *Scenedesmus obliquus* cultivated in municipal wastewater. Luna et al. [94] observed a modification change in the disposition of tilacoids in the cyanobacteria *Synechocystis aquatilis* induced by MF (37.7–44.3 mT), varying the distribution and distances between the tilacoids and the tilacoidal membranes. Other possible explanations have been reported by Santos et al. [89]. The studies have focused on cultivating different microalgae with MF application and have verified what the observed effects are. Microalgae that have already been evaluated are *Nannochloropsis oculata* [95], *Chlorella fusca* [96,97], *Spirulina* sp. [98,99], and other microalgae species cited in Table 4.

The observed effects are different depending on the conditions of the MF application. Luna et al. [94] observed that with *Chlorella vulgaris*, the continuous MF application furthers the carbohydrate, lipid, and protein accumulation in the exponential growth phase and lipids in the stationary phase. Deamici et al. [96] verified that 60 mT applied throughout the cultivation increased by 20.5% and 24.8% in terms of biomass and carbohydrate content, respectively. Han et al. [100] observed that 0.5 T stimulated 12% biomass and 10% lipid productivity when MF was applied throughout the cultivation. Chu et al. [95] verified the effects of MF and nitrate concentration on the growth of *Nannochloropsis oculate*. When using 20 mT and 150 mg L^−1^ nitrate, maximum specific growth rate and maximum lipid productivity increased by 166% and 103%, respectively. Huo et al. [101] observed that 30 mT may affect biochemical composition of *Tribonema* sp. and enhance the oil accumulation.

## 4. Possible Mechanisms of Magnetopriming

**Figure 2:**
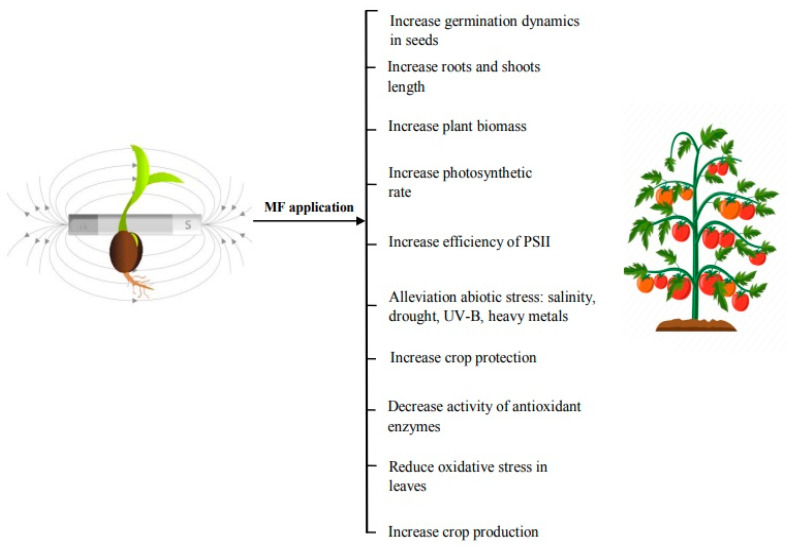
The various morphological, physiological, and biochemical effects of magnetic field on the plants.

In biological systems, a number of hypotheses have been proposed for the mechanisms of MF perception and responses, amongst them ion cyclotron resonance model, parametric resonance model, coherent quantum excitations, and free-radical and other spin mechanisms currently receiving more attention [12]. Enzyme-catalyzed reactions involving intermediates with free radical pairs are also called “radical pair mechanism” (RPM). The modulation of the singlet–triplet conversion rate of the free radical pairs is affected by a weak magnetic field. In the process of mitochondrial respiratory chain, porphyrins produce free radicals, which may also be influenced by an external magnetic field as it affects singlet–triplet conversions. The RPM is currently the only physically plausible mechanism indicating the role of cryptochrome as a candidate for magneto reception that results in generation of flavin-trytophan radical pairs [35]. Cryptochromes are light flavor protein receptors and are thought to be involved in plants’ magneto perception because they can form radical pairs of reduced flavine adenine dinucleotide (FADH2) and tryptophan residue in the protein structure after exposure to blue light [103,104]. On the basis of the ferrimagnetism hypothesis, perception of SMF by plants is achieved through its direct effect on iron particles, mineral iron compounds (e.g., Fe_3_O_4_ and Fe_3_S_4_), and iron-containing proteins [104,105]. Phytoferritin occurs in plant cells as crystalline magnetite (Fe_3_O_4_), ε-Fe_2_O_3_, and hematite (α-Fe_2_O_3_) [106], and may interact stronger with the magnetic fields than with the diamagnetic or paramagnetic materials. These particles can also affect the superoxide-generated free radicals [107]. There are several reports that indicate that MF can cause overproduction of ROS and initiation of oxidative stress. Moreover, the influence of SMF also altered the activities of enzymatic antioxidants or the expression of their genes [104,108,109,110]. The other theory of “ion cyclotron resonance” revolves around the fact that ions should circulate in a plane perpendicular to an external magnetic field with their Lamor frequencies, which can interfere with an alternating electromagnetic field [34,111]. The biological effects may be elucidated as an interaction between MF and ionic current in the plant embryo cell membrane, which causes alterations in both ionic concentrations and osmotic pressure on both sides of the membrane [112]. Changes in water uptake mechanisms are caused by alterations in the ionic fluxes across the cell membrane [66]. Imbibitions of magnetically treated seeds showed faster hydration of macromolecules and membranes and greater activities of enzymes such as α-amylase and nitrate reductase during seed germination, which are responsible for quicker germination of magnetoprimed seeds as compared to unexposed seeds [45,48,113,114,115,116,117]. The various morphological, physiological, and biochemical effects of magnetic field seed pretreatment on the plants are represented in Figure 2.

## 5. Conclusions and Prospects

To increase seed germination, crop productivity, and development, a proper combination of MF intensity and time exposure is essential. Many studies have proved that its positive impacts can improve seed germination, root and shoot length, the absorption of the water and CO_2_, the content of the photosynthetic pigments, and finally the increase of the agricultural production even under abiotic stresses. On the other hand, other researchers have shown that the aforementioned parameters have not been improved, rather, they have been declined since, and the MF causes growth inhibition of the plants. The mechanism by which plants perceive MFs and regulate the signal transduction pathway is not fully understood. It has been suggested that MF perception/signaling in plants is mediated by the blue light photoreceptors—cryptochromes. It has also been found that ROS and NO are the signaling molecules for magnetopriming-induced seed germination. However, this aspect of magneto biology still deserves in-depth investigation, as well as the potential genotoxic side effects of MFs. All these works have highlighted the need for more studies to extend our knowledge on the molecular mechanisms involved in fastening seed germination, higher seedling vigor, and enhancing photosynthetic capacity of magneto-primed plants under abiotic stresses. In general, it seems that in spite of all the efforts and studies performed on the MFs, there is still a gap in human knowledge, and thus further experiments are needed.

## Figures and Tables

**Table 1:** Application of magnetic field in seed germination.

Plant Species	Plant Organ	MF Intensity	Effects	References
*Vigna radiata* (Linn.) Wilczek.	Seeds	87–226 mT SMF	Increase in time and in mean germination rate, as well as in water uptake by seeds according to increasing intensity of magnetic field	[6]
*Passiflora edulis* Sims	Seeds	200 mT SMF	Stimulates seed germination, emergence, and vigor of seedlings	[8]
*Cucumis sativus* L.	Seeds	200 mT SMF	Superiority germinative and increased activities of hydrolytic enzymes, reactive oxygen species, and antioxidant enzyme system during germinating seeds	[17]
*Glycine max* (Linn.) Merr.	Seeds Seedlings	150–200 mT SMF	Increase of photosynthetic rate, seed germination, crop yield, pigment synthesis, biomass, nitrogen metabolism, and root nodules	[16,26,45]
*Cicer arietinum* L.	Seeds, Seedlings	50–150 mT SMF	Enhanced performance in rate and speed of seed germination, superiority in the seedling growth, and in functional root parameters	[20,36]
*Triticum aestivum* L.	Seeds Seedlings	4–7 mT SMF	Enhancement of seed germination, seedling growth	[25]
*Solanum lycopersicum* Mill.	Seeds	50–332 mT SMF	Increase in germination rate, promoved biochemical and molecular changes involved in homeostasis of hydrogen peroxide (H_2_O_2_) promoting the seed vigor	[19,38]
*Zea mays* L.	Seeds Seedlings	200 mT SMF	Enhancement of seed germination, seedling growth, a-amylase, protease, and free-radicals	[21,37]
*Raphanus sativus* L.	Seeds	8–20 mT SMF	Increased the rate and the vigor index of germination	[39]
*Capsicum annuum* L.	Seeds Seedlings	57–60 mT SMF	Enhancement of seed germination, seedling growth, and yield and fruit quality	[46]
*Hordeum vulgare* L.	Seeds Seedlings	35 mT SMF	Enhancement of seed germination and seedling establishment under normal or saline stress conditions	[47]
*Helianthus annuus* L.	Seeds Seedlings	50–200 mT SMF	Increased the speed of germination and induced the early vigor of seedlings	[48]
*Oryza sativa* L.	Roots Seeds	125–250 mT MF	Increased root and stem length Increased germination dynamics in seeds	[49]
*Phaseolus vulgaris* L.	Seeds Seedlings	4–7 mT 130 mT MF	Enhancement of seed germination and seedling growth, and promoted mitotic activity in meristematic plant cells Increase of glutathione peroxidase (GPOX) activity in leaves	[25,50]

**Table 2:** Functions of magnetically treated water in soil and plants.

Plant Species	Method	Effect	Reference
*Solanum melomgena* L. *Vicia faba* L. *Solanum lycopersicon* L.	MTW	Neutralizing soil pH value The yield gain per water unit was 2.47% on average for the three crops	[57]
*Lens culinaris* Medik	110 mT MW	Significantly enhanced the activity of APX and decreased the activity of SOD	[67]
*Allium cepa* L.	MTW (120–150 mT)	Increased the amount of phosphorus in leaves Lowered soil alkalinity	[59]
*Citrus sinensis* [L.] Osbeck	MTW	Seeds with low vigor can be invigorated 13.3% increase in germination	[24]
*Triticum aestivum* L.	MTW MF-treated seeds	Decreased the downward mobility of the mineral compounds	[10]
*Apium graveolens* L. *Pisum sativum* L.	MTW (136 mT)	MTW mitigated the adverse effects of drought in the *Moringa* species by improving the Na^+^/K^+^ ratio	[23]
*Moringa oleifera* Lam. *M. peregrina* (Forssk. Fiori)	MTW (30 mT)	Increased efficiency of salty water and enhanced growth criteria	[63]
*Cynara scolymus* L.	MTW (300 mT)	Increased photosynthetic pigments significantly Increased nutrient uptake efficiency (N, P, K, Fe, Mn, Zn, and Cu)	[65]
Fragaria × ananassa *Solanum lycopersicum* Lam.	MTW	Increased protein content (28.92%), alpha amylase (11.36%), and protease activities (14.76%) over the control	[61]
*Brassica rapa* L. var. glabra Regel	MTW (211 mT)	Decreased EC and TDS by 15.60% after 300 min The soil-soluble Na^+^ significantly decreased from 15.53 to 8.57 mEq/L	[68]
*Cucumis sativus* L. *Cucumis melo* L.	MTW (40 mT) MF (40 mT) treated seeds	A higher nutrient uptake, reduction of toxicitym, and sodium concentration in the aerial parts Increased the amount of phosphorus in leaves, lowering soil alkalinity	[62,69]

**Table 3:** The mitigation effect of magnetic fields (MFs) in abiotic stress.

Plant Species	Abiotic Stress	Adaptive Response of Plants by Magnetopriming	References
*Vigna radiata* L.	Cadmium stress	Increased growth, photosynthetic pigments, efficiency of PSII, photosynthesis	[84]
*Zea mays* L.	Salt stress	Seedling vigor, increased activities of α amylase and protease enzymes; increased growth, PSII efficiency, photosynthesis, and yield	[21,85]
*Cicer arietinum* L.	Salt stress	Improvement in germination rate and growth root and shoot; greater water uptake and increased activities of α amylase and protease enzymes	[20]
*Glycine max* (Linn.) Merr.	Water stress	Increased growth, photosynthetic pigments, efficiency of PSII, photosynthesis, and crop yield	[86]
*Glycine max* (Linn.) Merr.	Salt stress	Increased the seed germination	[26]
*Glycine max* (Linn.) Merr.	UV-B stress	Increased growth, efficiency of PSII, photosynthesis, and carbonic anhydrase/nitrogenase activities; higher DNA, RNA, protein, and nitric oxide content in leaves; and reduced ROS and antioxidant defense system, along with improved crop yield	[27,88]
*Glycine max* (Linn.) Merr.	Salt stress	Involvement of nitrate reductase in nitric oxide production in alleviation of salt stress during seed germination	[45]

**Table 4:** Effect of magnetic field on algae cultivation.

Plant Species	Type of Algae	MF Intensity	Effects	References
*Nannochloropsis oculata*	Green algae	20 mT	Growth increased by 20.5% Increased carbohydrate concentration 24.8%	[95]
*Spirulina* sp.	Green algae	25 mT	Enhanced growth in outdoor culture system	[98]
*Tribonema* sp.	Yellow-green algae	30 mT	Improved the oil accumulation Improved the productivity of biomass, protein, and carbohydrate	[101]
*Haematococcus pluvialis*	Red algae	30 mT	Increased growth, pigment synthesis, and cell division	[94]
*Arthrospira platensis*	Green algae	30 mT	Enhanced the PSII performance Enhanced growth by 49% and carbohydrate by 15%	[99]
*Scenedesmus obliquus*	Green algae	0.1 T	Stimulated oxygen production and algal growth Increase in chlorophyll-a by 11.5%	[93]
*Chlorella pyrenoidosa*	Green algae	0.5 T	Increase of the lipid product by 10% Increase in useful bacteria, active oxygen, and biomass	[100]
*Chlorella fusca Chlorella kessleri*	Green algae	60 mT 30 mT	Growth increased, increased biomass concentration, stimulated cell growth and bio-compound synthesis, effect hormetic of MF on cells Increase in protein by 8.9% and lipid synthesis by 13.1%	[96,102]
